# Supplementary material for: Cost-effectiveness of monitoring and liver cancer surveillance among patients with inactive chronic hepatitis B
Source: PLoS One. 2025 Jan 22;20(1):e0313898. doi: 10.1371/journal.pone.0313898 (PMC11753660; doi:10.1371/journal.pone.0313898)
Supplement: S1 File — (DOCX) [file pone.0313898.s001.docx]

**Supporting Information: Cost-Effectiveness of Monitoring and Liver Cancer Surveillance among Patients with Inactive Chronic Hepatitis B**

[S1 Table. Natural History Transition Estimates 2](#_Toc174536862)

[S2 Table. Treatment transition estimates 5](#_Toc174536863)

[S3 Table. Alternative definition of the percent monitoring assuming all are eligible for monitoring, but receive monitoring at irregular intervals compared with current practice for 1,000,000 adults with inactive CHB 8](#_Toc174536864)

[S4A Table. Lifetime per person cost-effectiveness outcomes for increase monitor only scenario compared to current practice 9](#_Toc174536865)

[S4B Table. Lifetime per person cost-effectiveness outcomes for increase treatment only scenario compared to current practice 10](#_Toc174536866)

[S5 Table. Outcomes in life years for 100,000 adults with inactive CHB with HCC risk greater or equal to 0.55%/year 11](#_Toc174536867)

[Appendix only references: 12](#_Toc174536868)

#

# S1 Table. Natural History Transition Estimates

| Transition (per year) | Natural History Estimate | Range | Reference |
| --- | --- | --- | --- |
| From Inactive HBeAg-negative CHB |  |  |  |
| To HBsAg loss | 1.25% | (0.98-1.54) | [1] |
| To HBeAg-negative active CHB |  |  |  |
| Age-group < 30 | 0.89% | (0.71-1.06) | [2] |
| Age-group 30-39 | 1.53% | (1.23-1.83) | [2] |
| Age-Group 40-49 | 2.14% | (1.71-2.56) | [2] |
| Age-Group 50+ | 1.51% | (1.21-1.81) | [2] |
| To Cirrhosis |  |  |  |
| Age-group <30 | 0.03% | (0.024-0.036) | [2] |
| Age-group 30-39 | 0.25% | (0.20-0.30) | [2] |
| Age-Group 40-49 | 0.53% | (0.43-0.63) | [2] |
| Age-Group 50+ | 0.71% | (0.57-0.85) | [2] |
| To HCC | 0.17% | (0.02-0.62) | [3] |
| From HBeAg-positive immunotolerant CHB |  |  |  |
| To HBeAg-positive immune active CHB | 1.26% | (1.01-1.51) | [4] |
| From HBeAg-positive immune active CHB |  |  |  |
| To HBsAg loss | 0.60% | (0.3-0.9) | [5] |
| To Cirrhosis | 1.60% | (1.3-1.9) | [6] |
|  |  |  |  |
| To HCC | 1.47% | (0.40-2.55) | [7] |
|  |  |  |  |
| To HBV-related Death | 0.11% | (0.09-0.14) | [7] |
| To HBeAg-positive inactive CHB | 7% | (4.0-10.0) | [8] |
| From HBeAg-negative active CHB |  |  |  |
| To HBsAg loss | 0.60% | (0.3-0.9) | [5] |
| To Active HBeAg-positive (reversion) | 0.16% | (0.08-0.24) | [9-11] |
| To Cirrhosis | 2.80% | (1.3-4.3) | [6] |
| To HCC | 0.72% | (0.21-1.23) | [7] |
|  |  |  |  |
| To HBV-related Death | 0.11% | (0.09-0.14) | [7] |
| To Inactive | 1.60% | (0.0-6.0) | [8] |
| From Compensated Cirrhosis |  |  |  |
| To HBsAg loss | 0.60% | (0.3-0.9) | [5] |
| To Decompensated Cirrhosis | 3.90% | (1.95-5.85) | [12] |
| To HCC | 3.16% | (2.58-3.74) | [7] |
| To HBV-related Death | 4.89% | (3.16-6.63) | [7] |
| To Viral Suppression | 6.30% | (3.15-9.45) | [13] |
| From Decompensated Cirrhosis |  |  |  |
| To Liver Transplantation | 1.20% | (1.0-3.0) | [14] |
| To HCC | 7.10% | (3.55-10.65) | [12] |
|  |  |  |  |
| To HBV-related Death | 15% | (7.50-22.5) | [12] |
| From HCC |  |  |  |
| To Liver Transplantation | 7% | (5.0-9.0) | [14] |
| To HBV-related Death | 42.5% |  | [15] |
| From Viral Suppression Cirrhosis |  |  |  |
| To HBsAg loss | 1% | (0.5-1.5) | [5] |
| To HCC | 1.58% | (1.29-1.87) | [7] |
| To HBV-related Death | 2.44% | (1.58-3.31) | [7] |
| From Liver Transplantation Decompensated Cirrhosis |  |  |  |
| To HBV-related death year 1 | 17% | (8.5-48.0) | [16] |
| To HBV-related death year 2+ | 2.50% | (1.25-24.0) | [16] |
| From Liver Transplantation HCC |  |  |  |
| To HBV-related death year 1 | 16% | (8.0-48.0) | [16] |
| To HBV-related death year 2+ | 2% | (2.0-25.0) | [16] |
| From HBsAg loss |  |  |  |
| To Cirrhosis | 0.28% | (0.14-0.42) | [2, 17] |
| To HCC | 0.09% | (0.045-0.136) | [18] |
| Gender |  |  |  |
| Relative Progression Rates for females* | 0.5 | (0.25-1.0) | [19-21] |
| Fraction of chronic HBV cases that are Male | 65% | (0.50-0.80) | [22] |

A 50% reduction in disease progression estimates was applied for females

Abbreviations: CHB, chronic hepatitis B; HBeAg, hepatitis B e antigen; HBsAg, hepatitis B surface antigen; HBV, hepatitis B virus; HCC, hepatocellular carcinoma

#

# S2 Table. Treatment transition estimates

| Transition (per year) | Treatment Estimate | Range | Reference |
| --- | --- | --- | --- |
| From HBeAg-positive immune active CHB |  |  |  |
| To HBsAg loss | 3% | (1.5-4.5) | [23] |
| To Cirrhosis | 0 | 0 | assumption |
|  |  |  |  |
| To HCC | 0.44% | (0.12-0.765) | (70% reduction)[24, 25] |
|  |  |  |  |
| To HBV-related Death | 0 | 0 | assumption |
| To Drug Resistance | 0.01% | (0.0-0.01) | [26-28] |
| To Viral Suppression | 76% | (65.0-85.0) | [23] |
| From HBeAg-negative active CHB |  |  |  |
| To HBsAg loss | 1% | (0.5-1.5) | [23] |
| To Cirrhosis | 0 | 0 | assumption |
| To HCC | 0.22% | (0.063-0.369) | (70% reduction)[24, 25] |
|  |  |  |  |
| To HBV-related Death | 0 | 0 | assumption |
| To Drug Resistance | 0.01% | (0.0-0.01) | [26-28] |
| To Viral Suppression | 93% | (65.0-99.0) | [23] |
| From Compensated Cirrhosis |  |  |  |
| To HBsAg loss | 1.70% | (0.85-2.55) | [29] |
| To Decompensated Cirrhosis | 1.80% | (0.90-2.70) | (50% reduction) |
| To HCC | 1.60% | (1.25-1.75) | (50% reduction) [30] |
| To HBV-related Death | 2.40% | (1.58-3.30) | (50% reduction) |
| To Viral Suppression | 78% | (65.0-78.0) | [30] |
| To Drug Resistance | 0.01% | (0.0-0.01) | [26-28] |
| From Decompensated Cirrhosis |  |  |  |
| To Liver Transplantation | 1.20% | (0.60-1.80) | [14] |
| To HCC | 3.50% | (1.75-5.25) | (50% reduction) [30] |
|  |  |  |  |
| To HBV-related Death | 7.50% | (3.75-11.25) | (50% reduction) |
| To Viral Suppression | 78% | (65.0-78.0) | [30] |
| To Drug Resistance | 0.01% | (0.0-0.01) | [26-28] |
| From HCC |  |  |  |
| To Liver Transplantation | 7% | (5.0-9.0) | [14] |
| To HBV-related Death | 31.1% | (26.4-31.1) | (27% reduction)[31] |
| From Viral Suppression CHB |  |  |  |
| To HBsAg loss | 1.50% | (0.07-2.2) | [23] |
| To HCC | 0.06% | (0.03-0.09) | (70% reduction)[24, 25] |
|  |  |  |  |
| From Viral Suppression Cirrhosis |  |  |  |
| To HBsAg loss | 1.50% | (0.07-2.2) | [23] |
| To HCC | 0.80% | (0.40-1.20) | (50% reduction) |
| To HBV-related Death | 1.20% | (0.60-1.80) | (50% reduction) |
| From Viral Suppression Decompensated Cirrhosis |  |  |  |
| To HCC | 3% | (1.5-4.5) | [32] |
| To HBV-related Death | 6.10% | (3.05-9.15) | [32] |
| From Liver Transplantation for Decompensated Cirrhosis |  |  |  |
| To HBV-related death year 1 | 17%-32% | (8.5-48.0) | [16] |
| To HBV-related death year 2+ | 2.50% | (1.25-24.0) | [16] |
| From Liver Transplantation for HCC |  |  |  |
| To HBV-related death year 1 | 16%-39% | (8.0-48.0) | [16] |
| To HBV-related death year 2+ | 2% | (2.0-25.0) | [16] |
| Gender |  |  |  |
| Relative Progression Rates for females* | 0.5 | (0.25-1.0) | [19-21] |
| Fraction of chronic HBV cases that are Male | 65% | (0.50-0.80) | [22] |

A 50% reduction in disease progression estimates was applied for females

# S3 Table. Alternative definition of the percent monitoring assuming all are eligible for monitoring, but receive monitoring at irregular intervals compared with current practice for 1,000,000 adults with inactive CHB

| **Scenario** | **Cost** | **QALYs** | **ICER** | **Cirrhosis** | **Decompensated Cirrhosis** | **HCC** | **HBV Deaths** |
| --- | --- | --- | --- | --- | --- | --- | --- |
| **Current Practice (CP)** | $180,249,693,212 | 19,425,584 | - | 122,935 | 3,085 | 55,350 | 63,189 |
| **Increased M&T** | $181,974,224,211 | 19,494,702 | $24,951 | 110,804 | 1,843 | 52,738 | 60,486 |
| **Difference** | $1,724,530,999 | 69,118 |  | -12,130 | -1,242 | -2,612 | -2,703 |

# S4A Table. Lifetime per person cost-effectiveness outcomes for increase monitor only scenario compared to current practice

| **Scenario** | **Cost** | **QALYs** | **ICER** | **Cirrhosis** | **Decompensated Cirrhosis** | **HCC** | **HBV Deaths** |
| --- | --- | --- | --- | --- | --- | --- | --- |
| **Current Practice (CP)**  37% monitored and 59% treated | $ 182,759 | 18.918 | - | 17.21% | 2.09% | 8.47% | 12.10% |
| **Increased Monitor**  90% monitored and 59% treated | $ 182,578 | 19.368 | Cost-saving | 12.62% | 0.59% | 6.02% | 7.40% |
| **Difference** | -$ 181 | +0.450 | -$ 404 | -4.59% | -1.50% | -2.45% | -4.70% |

# S4B Table. Lifetime per person cost-effectiveness outcomes for increase treatment only scenario compared to current practice

| **Scenario** | **Cost** | **QALYs** | **ICER** | **Cirrhosis** | **Decompensated Cirrhosis** | **HCC** | **HBV Deaths** |
| --- | --- | --- | --- | --- | --- | --- | --- |
| **Current Practice (CP)**  37% monitored and 59% treated | $ 182,759 | 18.918 | - | 17.21% | 2.09% | 8.47% | 12.10% |
| **Increased Treatment**  37% monitored and 80% treated | $ 182,388 | 19.003 | Cost-saving | 16.04% | 1.90% | 7.97% | 11.30% |
| **Difference** | -$ 371 | +0.085 | -$ 4,375 | -1.17% | -0.19% | -0.50% | -0.80% |

# S5 Table. Outcomes in life years for 100,000 adults with inactive CHB with HCC risk greater or equal to 0.55%/year

| **Scenario** | **Cost** | **Life Years** |
| --- | --- | --- |
| **Increased M&T** | $182,579,000,000 | 1,940,500 |
| **Increased M&T plus HCC surveillance with biannual US+AFP** | $183,463,000,000 | 1,940,900 |
| **Difference** | $884,000,000 | +400 |

#

## Appendix only references:

1. Zhou K, Contag C, Whitaker E, Terrault N. Spontaneous loss of surface antigen among adults living with chronic hepatitis B virus infection: a systematic review and pooled meta-analyses. Lancet Gastroenterol Hepatol. 2019;4(3):227-38. Epub 2019/01/27. doi: 10.1016/S2468-1253(18)30308-X. PubMed PMID: 30679109; PubMed Central PMCID: PMCPMC6541384.

2. Chu CM, Liaw YF. HBsAg seroclearance in asymptomatic carriers of high endemic areas: appreciably high rates during a long-term follow-up. Hepatology. 2007;45(5):1187-92. Epub 2007/04/28. doi: 10.1002/hep.21612. PubMed PMID: 17465003.

3. Raffetti E, Fattovich G, Donato F. Incidence of hepatocellular carcinoma in untreated subjects with chronic hepatitis B: a systematic review and meta-analysis. Liver Int. 2016;36(9):1239-51. doi: 10.1111/liv.13142. PubMed PMID: 27062182.

4. Hui CK, Leung N, Yuen ST, Zhang HY, Leung KW, Lu L, et al. Natural history and disease progression in Chinese chronic hepatitis B patients in immune-tolerant phase. Hepatology. 2007;46(2):395-401. Epub 2007/07/14. doi: 10.1002/hep.21724. PubMed PMID: 17628874.

5. Ahn SH, Park YN, Park JY, Chang HY, Lee JM, Shin JE, et al. Long-term clinical and histological outcomes in patients with spontaneous hepatitis B surface antigen seroclearance. J Hepatol. 2005;42(2):188-94. doi: 10.1016/j.jhep.2004.10.026. PubMed PMID: 15664243.

6. Fattovich G, Bortolotti F, Donato F. Natural history of chronic hepatitis B: special emphasis on disease progression and prognostic factors. J Hepatol. 2008;48:335-52.

7. Thiele M, Gluud LL, Fialla AD, Dahl EK, Krag A. Large variations in risk of hepatocellular carcinoma and mortality in treatment naive hepatitis B patients: systematic review with meta-analyses. PLoS One. 2014;9(9):e107177. doi: 10.1371/journal.pone.0107177. PubMed PMID: 25225801; PubMed Central PMCID: PMCPMC4167336.

8. Kanwal F, Gralnek IM, Martin P, Dulai GS, Farid M, Spiegel BM. Treatment alternatives for chronic hepatitis B virus infection: a cost-effectiveness analysis. Ann Intern Med. 2005;142(10):821-31. PubMed PMID: 15897532.

9. Yang HI, Hung HL, Lee MH, Liu J, Jen CL, Su J, et al. Incidence and determinants of spontaneous seroclearance of hepatitis B e antigen and DNA in patients with chronic hepatitis B. Clin Gastroenterol Hepatol. 2012;10(5):527-34 e1-2. doi: 10.1016/j.cgh.2011.12.019. PubMed PMID: 22178461.

10. Hsu YS, Chien RN, Yeh CT, Sheen IS, Chiou HY, Chu CM, et al. Long-term outcome after spontaneous HBeAg seroconversion in patients with chronic hepatitis B. Hepatology. 2002;35(6):1522-7. Epub 2002/05/25. doi: S0270913902866903 [pii]

10.1053/jhep.2002.33638. PubMed PMID: 12029639.

11. Pungpapong S, Kim WR, Poterucha JJ, editors. Natural history of hepatitis B virus infection: an update for clinicians. Mayo clinic proceedings; 2007: Elsevier.

12. Lin X, Robinson NJ, Thursz M, Rosenberg DM, Weild A, Pimenta JM, et al. Chronic hepatitis B virus infection in the Asia-Pacific region and Africa: review of disease progression. J Gastroenterol Hepatol. 2005;20(6):833-43. PubMed PMID: 15946129.

13. Chen YC, Chu CM, Liaw YF. Age-specific prognosis following spontaneous hepatitis B e antigen seroconversion in chronic hepatitis B. Hepatology. 2010;51(2):435-44. Epub 2009/11/18. doi: 10.1002/hep.23348. PubMed PMID: 19918971.

14. Organ Procurement and Transplantation Network. [cited 2016 July ]. Available from: <https://optn.transplant.hrsa.gov/data/view-data-reports/build-advanced/>.

15. Ding J, Wen Z. Survival improvement and prognosis for hepatocellular carcinoma: analysis of the SEER database. BMC Cancer. 2021;21(1):1157. Epub 2021/10/31. doi: 10.1186/s12885-021-08904-3. PubMed PMID: 34715816; PubMed Central PMCID: PMCPMC8555190.

16. Burra P, Germani G, Adam R, Karam V, Marzano A, Lampertico P, et al. Liver transplantation for HBV-related cirrhosis in Europe: an ELTR study on evolution and outcomes. J Hepatol. 2013;58(2):287-96. doi: 10.1016/j.jhep.2012.10.016. PubMed PMID: 23099188.

17. Chu CM, Liaw YF. Incidence and risk factors of progression to cirrhosis in inactive carriers of hepatitis B virus. Am J Gastroenterol. 2009;104(7):1693-9. Epub 2009/05/21. doi: 10.1038/ajg.2009.187. PubMed PMID: 19455130.

18. Liu J, Yang HI, Lee MH, Lu SN, Jen CL, Batrla-Utermann R, et al. Spontaneous seroclearance of hepatitis B seromarkers and subsequent risk of hepatocellular carcinoma. Gut. 2014;63(10):1648-57. doi: 10.1136/gutjnl-2013-305785. PubMed PMID: 24225939.

19. Le A, Toy M, Yang HI, Trinh HN, Zhang JQ, Wong C, et al. Age and gender-specific disease progression rates to cirrhosis and hepatocellular carcinoma in treated and untreated patients with chronic hepatitis B. AASLD; Washington D.C2017.

20. Guy J, Peters MG. Liver disease in women: the influence of gender on epidemiology, natural history, and patient outcomes. Gastroenterol Hepatol (N Y). 2013;9(10):633-9. PubMed PMID: 24764777; PubMed Central PMCID: PMCPMC3992057.

21. Cohen E, Tran TT. Hepatitis B in the Female Population. Gastroenterol Clin North Am. 2016;45(2):359-70. doi: 10.1016/j.gtc.2016.02.011. PubMed PMID: 27261904.

22. Patel EU, Thio CL, Boon D, Thomas DL, Tobian AAR. Prevalence of Hepatitis B and Hepatitis D Virus Infections in the United States, 2011-2016. Clin Infect Dis. 2019;69(4):709-12. Epub 2019/01/04. doi: 10.1093/cid/ciz001. PubMed PMID: 30605508; PubMed Central PMCID: PMCPMC6669285.

23. Terrault NA, Bzowej NH, Chang KM, Hwang JP, Jonas MM, Murad MH, et al. AASLD guidelines for treatment of chronic hepatitis B. Hepatology. 2016;63(1):261-83. doi: 10.1002/hep.28156. PubMed PMID: 26566064.

24. Papatheodoridis GV, Chan HL, Hansen BE, Janssen HL, Lampertico P. Risk of hepatocellular carcinoma in chronic hepatitis B: assessment and modification with current antiviral therapy. J Hepatol. 2015;62(4):956-67. doi: 10.1016/j.jhep.2015.01.002. PubMed PMID: 25595883.

25. Nguyen MH, Yang HI, Le A, Henry L, Nguyen N, Lee MH, et al. Reduced Incidence of Hepatocellular Carcinoma in Cirrhotic and Noncirrhotic Patients With Chronic Hepatitis B Treated With Tenofovir-A Propensity Score-Matched Study. J Infect Dis. 2019;219(1):10-8. Epub 2018/07/10. doi: 10.1093/infdis/jiy391. PubMed PMID: 29982737.

26. Heathcote EJ, Marcellin P, Buti M, Gane E, De Man RA, Krastev Z, et al. Three-year efficacy and safety of tenofovir disoproxil fumarate treatment for chronic hepatitis B. Gastroenterology. 2011;140(1):132-43. Epub 2010/10/20. doi: S0016-5085(10)01499-X [pii]

10.1053/j.gastro.2010.10.011. PubMed PMID: 20955704.

27. Lok AS, McMahon BJ, Brown RS, Jr., Wong JB, Ahmed AT, Farah W, et al. Antiviral therapy for chronic hepatitis B viral infection in adults: A systematic review and meta-analysis. Hepatology. 2016;63(1):284-306. doi: 10.1002/hep.28280. PubMed PMID: 26566246.

28. Tenney DJ, Rose RE, Baldick CJ, Pokornowski KA, Eggers BJ, Fang J, et al. Long-term monitoring shows hepatitis B virus resistance to entecavir in nucleoside-naive patients is rare through 5 years of therapy. Hepatology. 2009;49(5):1503-14. PubMed PMID: 19280622.

29. Buti M, Fung S, Gane E, Afdhal NH, Flisiak R, Gurel S, et al. Long-term clinical outcomes in cirrhotic chronic hepatitis B patients treated with tenofovir disoproxil fumarate for up to 5 years. Hepatol Int. 2015;9(2):243-50. doi: 10.1007/s12072-015-9614-4. PubMed PMID: 25788199; PubMed Central PMCID: PMCPMC4387268.

30. Wong GL, Chan HL, Mak CH, Lee SK, Ip ZM, Lam AT, et al. Entecavir treatment reduces hepatic events and deaths in chronic hepatitis B patients with liver cirrhosis. Hepatology. 2013. Epub 2013/02/08. doi: 10.1002/hep.26301. PubMed PMID: 23389810.

31. Thein HH, Campitelli MA, Yeung LT, Zaheen A, Yoshida EM, Earle CC. Improved Survival in Patients with Viral Hepatitis-Induced Hepatocellular Carcinoma Undergoing Recommended Abdominal Ultrasound Surveillance in Ontario: A Population-Based Retrospective Cohort Study. PloS one. 2015;10(9):e0138907. Epub 2015/09/24. doi: 10.1371/journal.pone.0138907. PubMed PMID: 26398404; PubMed Central PMCID: PMCPMC4580446.

32. Jang JW, Choi JY, Kim YS, Woo HY, Choi SK, Lee CH, et al. Long-term effect of antiviral therapy on disease course after decompensation in patients with hepatitis B virus-related cirrhosis. Hepatology. 2015;61(6):1809-20. doi: 10.1002/hep.27723. PubMed PMID: 25627342.
