# Supplementary figures and images for: Cost-effectiveness of monitoring and liver cancer surveillance among patients with inactive chronic hepatitis B
Source: PLoS One. 2025 Jan 22;20(1):e0313898. doi: 10.1371/journal.pone.0313898 (PMC11753660; doi:10.1371/journal.pone.0313898)

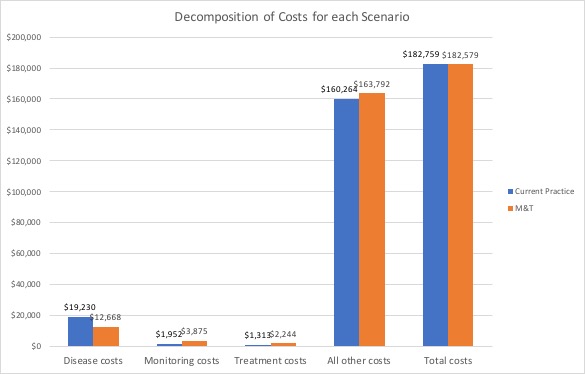

Supplement: S1 Fig — * “All other costs” are age-specific background medical costs (non-HBV-related, such as for heart disease). (JPG) [file pone.0313898.s002.jpg]

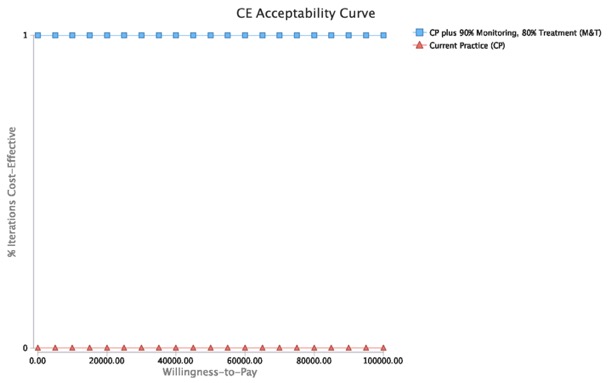

Supplement: S2 Fig — This figure shows the probability that each strategy might be cost-effective (Y-axis) at particular values of willingness-to-pay for QALYs (X-axis). Because the CP plus M&T strategy is highly likely to lead to both savings in costs and improvement in QALYs, it is highly likely that CP plus M&T is cost-effective, regardless of the willingness to pay for QALYs. (JPG) [file pone.0313898.s003.jpg]

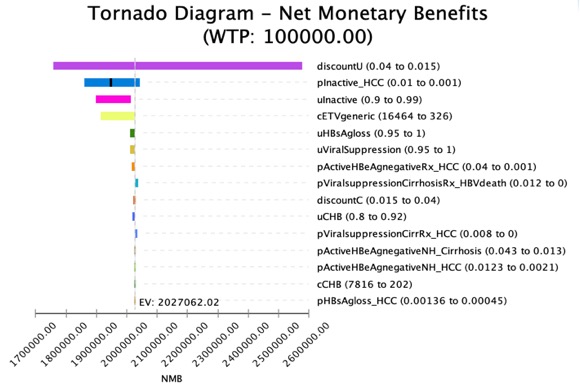

Supplement: S3 Fig — (JPG) [file pone.0313898.s004.jpg]
